# A Generalized Deep Learning Pipeline for Stain-Invariant Ultrastructural Segmentation in Peripheral Nerves

**DOI:** 10.3390/jimaging12060257

**Published:** 2026-06-10

**Authors:** Vitalijs Borisovs, Guido Cavaletti

**Affiliations:** 1 Experimental Neurology Unit, School of Medicine and Surgery, Università di Milano-Bicocca, 20900 Monza, Italy; guido.cavaletti@unimib.it; 2 Fondazione IRCCS San Gerardo dei Tintori, 20900 Monza, Italy

**Keywords:** Volume Electron Microscopy, deep learning segmentation, domain generalization, peripheral nerve ultrastructure, image standardization

## Abstract

Automated analysis of peripheral nerve ultrastructure is bottlenecked by heterogeneous electron microscopy (EM) datasets, where varying staining protocols and resolutions create domain shifts that confound deep learning. To address this, we developed a generalized segmentation pipeline. Using a custom pre-processing workflow (CLAHE and noise suppression) integrated into ZEISS Arivis Pro, we standardized inputs across three disparate domains: traditional osmium-based Palade, lanthanide-based “green” Uranyl-free method, and low-resolution Ellisman preparations. A U-Net trained on a highly constrained 15-image composite dataset achieved peak internal Intersection over Union (IoU) scores >0.95 for myelin and Schwann cells. Crucially, during open-world, zero-shot inference on an expanded independent testing cohort (N = 40), the model sustained robust Dice Similarity Coefficients of 0.854 for myelin and 0.597 for mitochondria. This demonstrates that integrating classical image standardization with deep learning effectively mitigates EM domain gaps, enabling comprehensive 3D multi-organelle reconstructions from challenging data. To ensure transparency and community utility, the pre-trained models and standardization scripts are provided in a public, open-access repository. Ultimately, this pipeline supports the transition to sustainable, non-toxic EM protocols and provides a robust pathway for unlocking historical clinical archives for automated organellomics.

## 1. Introduction

The peripheral nervous system architecture is a complex interplay of geometric precision and metabolic support. Quantifying its ultrastructure, specifically the morphology of myelin sheaths, the integrity of axonal mitochondria, and the cytoplasmic complexity of Schwann cells, is the gold standard for diagnosing and understanding the pathophysiology of the peripheral nervous system damage [1,2,3]. Metrics such as the g-ratio, defined as the axonal diameter relative to the total fiber diameter [4], along with the mitochondrial aspect ratio and the density of Schwann cell processes, are critical biomarkers that often precede gross anatomical degeneration or the onset of clinical symptoms [5,6].

However, the transition from manual, qualitative assessment to high-throughput, automated organellomics is currently bottlenecked by a fundamental lack of standardization in sample preparation [7,8]. Electron microscopy data is notoriously heterogeneous. The pixel intensity distribution and the overall contrast of an image are heavily dependent on the heavy metal staining protocol employed. Classical protocols, such as the Palade method using osmium tetroxide, rely on specific lipid–osmium interactions to provide high-contrast membrane definitions [9]. Conversely, emerging green alternatives like the lanthanide-based Uranyl-free staining generate fundamentally different image statistics [10], often emphasizing cytoskeletal elements over membranes [10,11]. Furthermore, research specimens are frequently constrained by processing times or archival conditions, which can result in lower-resolution images, mechanical damage, and increased artifacts compared to optimized experimental datasets [12].

This heterogeneity poses a significant challenge for standard deep learning segmentation pipelines. Convolutional neural networks are typically domain-specific [13,14]. A model trained on high-contrast, uranyl-stained images often fails when applied to uranyl-free or lower-resolution images due to the domain shift in the input data distribution [8]. While recent foundation models like Segment Anything have shown promise in general natural image segmentation [15], they often lack the zero-shot specificity required for the dense, cluttered ultrastructure of nerve tissue without extensive fine-tuning [16].

To address this, we propose a generalized deep learning pipeline. By integrating a custom Python (v3.14.5)-based pre-processing engine directly into the ZEISS Arivis Pro analysis ecosystem, we effectively bridge these disparate domains. This study demonstrates that a single model, trained on a composite of diverse datasets, can segment key ultrastructural components across highly variable staining protocols and image qualities, outperforming models trained on individual datasets.

While open-source biomedical frameworks like nnU-Net represent the current state-of-the-art in algorithmic segmentation [17], this study specifically targeted the deployment of a generalized model within a commercially available, user-friendly software ecosystem (ZEISS Arivis Pro v4.5.0). This ensures the resulting pipeline is immediately accessible to pathology laboratories without requiring advanced coding expertise.

## 2. Materials and Methods

### 2.1. Biological Specimens and Staining Protocols

Three distinct datasets of peripheral nerves were curated to represent the extremes of biological and technical variability encountered in neuropathology research. Detailed protocols regarding animal handling, tissue excision, and primary Serial Block-Face Scanning Electron Microscopy (SBF-SEM) image acquisition for the peripheral nerve samples have been previously described [18,19]. Briefly, samples from healthy Balb/c mice were imaged using a SBF-SEM Zeiss GeminiSEM 360 (Carl Zeiss S.p.A., Milan, Italy) equipped with a Volutome backscattered electron detector. Acquisitions were performed at an accelerating voltage of 1.75 kV, achieving an in-plane (X-Y) resolution of 6 nm/pixel and a slice thickness (Z-step) of 50 nm. For all multi-domain visualizations throughout this study, a fixed physical scale, indicated in the figure legend, was universally applied. Consequently, minor variations in the relative visual footprint of the scale bars across comparison panels reflect the differing native resolutions and fields of view inherent to the disparate imaging protocols.

The first dataset consists of 400 sequential slices of mouse caudal nerve samples processed using the classical Palade protocol with buffered osmium tetroxide [9]. This dataset represents the traditional research-grade standard, characterized by high membrane contrast and uniform preservation. The second dataset comprises 400 slices of murine sciatic nerve samples stained with Uranyl-free method, a synergistic mixture of Ytterbium(III) chloride and Phosphotungstic Acid [10]. This represents the green electron microscopy domain, characterized by unique textural properties with enhanced protein contrast but completely different gray-level histograms compared to osmium–uranium samples. The third dataset includes a targeted 100-slice volume of sciatic nerves processed utilizing the “gold-standard” Ellisman protocol with hot osmium–thiocarbohydrazide–osmium [12]. These samples represent a highly textured domain, characterized by high density but notably lower resolution and a reduced signal-to-noise ratio compared to the optimized experimental datasets.

### 2.2. Computational Workflow and Pre-Processing Script

The core of the computational workflow relies on a custom Python script integrated via the Python Application Programming Interface (API) within the ZEISS Arivis Pro environment. All raw image stacks were ingested into Arivis Pro, where the script executed a multi-stage preprocessing sequence to standardize disparate data domains before neural network inference.

To manage variant dynamic ranges, the script first applies Non-local Means Denoising (h = 10), which effectively suppresses stochastic noise while preserving the high-frequency edge information critical for detecting thin structures like the myelin sheath and endoplasmic reticulum (ER). Following denoising, the script performs global Z-score normalization across the volumetric stack, calculated as:

(1) $$\bar{x}=\;\frac{x\;-\;\mu }{\sigma \;+\;\varepsilon }$$

where *μ* and *σ* represent the global mean and standard deviation of the volume, and *ε* is a stability constant (1 × 10^−5^).

Local contrast is then optimized via Contrast Limited Adaptive Histogram Equalization (CLAHE) with a clip limit of 3.0 and a tile grid size of 8 by 8 [20]. This step flattens the domain gap by statistically aligning the input histograms of the high-contrast Palade images with the texture-heavy Uranyl-free and low-resolution data. Finally, the arrays are inverted and a 3 by 3 sharpening kernel is applied to accentuate membrane boundaries, ensuring the numerical stability and feature prominence required for robust deep learning inference.

### 2.3. Model Training and Evaluation Metrics

Model training employed an iterative, active-learning strategy natively supported by the ZEISS Arivis Pro environment, rather than a rigid a priori dataset partition. A foundational training set of 15 highly diverse, representative image planes, including data from all three sample preparations (Palade, Uranyl-free, and Ellisman), was curated. To manage computational resources and memory allocation during training and subsequent 3D volumetric rendering, raw datasets were cropped to relevant regions of interest (ROIs). Ground truth (GT) annotations for the structures of interest were manually generated across this composite set to capture the full spectrum of morphological and textural variability. Following initial training epochs, model predictions were evaluated, and additional targeted annotations were iteratively added to resolve challenging edge cases and refine the decision boundaries.

To ensure a rigorous and unbiased quantitative comparison between different models, training was constrained to an equivalent cumulative number of ground-truth annotations tailored to the morphological complexity of each target structure. Specifically, training utilized approximately 50 localized annotations for the myelin sheath (representing the minimum threshold for uniform macro-structures), 200 annotations for mitochondria, 450 annotations for the highly fragmented ER, and 200 annotations to capture the complex geometries of the Schwann cell cytoplasm and nuclei.

To establish a comparative baseline and isolate the impact of the pre-processing script, control models were trained on raw, unstandardized data for distinctly resolvable structures (myelin and mitochondria). However, due to the inherent lack of baseline contrast and target ambiguity in raw images, training specialist control models for fine micro-organelles like the ER was computationally unfeasible. Model training was executed using the proprietary Arivis Pro AI toolkit (Version 4.3.0). All models, including the baselines and the Generalized models, utilized a standard U-Net architecture and were initially trained for a fixed duration of 350 epochs, which represents the standard default configuration of the ZEISS Arivis architecture. The number of training epochs was gradually decreased during subsequent retraining iterations. To artificially expand the morphological variance of the training dataset and prevent overfitting, standard geometric data augmentations, including random rotations, reflections, and scaling, were applied natively to the sub-tiles during the default Arivis Pro deep learning training phase.

Regarding dataset partitioning and quantitative evaluation, initial performance metrics were derived from the models’ internal validation on the annotated datasets used during the active-learning phase. For the generalized models, this comprised a composite of 15 large-scale, high-resolution image planes from all three domains; for the baseline models, this comprised 15 high-resolution images strictly from the respective target domain. It is critical to note that in deep learning for high-resolution electron microscopy, each of these large-scale image planes is subsequently processed as thousands of discrete sub-tiles during convolutional training, providing a robust sample size for feature extraction. Because the Arivis Pro environment dynamically manages internal train-validation splits during its iterative cycles to optimize decision boundaries, these initial metrics reflect the models’ peak internal accuracy across the highly curated representative planes. To transition beyond internal metrics and rigorously verify true open-world generalization, the finalized Generalized pipeline was subjected to zero-shot inference on an expanded, independent testing cohort (N = 40 slices). Utilizing Z-axis stratified sampling across multiple domains, this standardized testing cohort allowed us to extract statistically robust performance metrics (e.g., standard deviations and confidence intervals) free from the technical variance and resolution-scaling artifacts present in initial smaller-scale evaluations.

Computational pre-processing, model training, and subsequent inference were performed on a dedicated workstation equipped with an NVIDIA Quadro RTX 4000 GPU (8 GB VRAM), 64 GB of RAM, and an Intel Xeon W-2275 CPU (3.30 GHz).

The performance of the segmentation models was evaluated using two rigorous spatial overlap metrics: the Dice Similarity Coefficient (DSC) and the Intersection over Union (IoU), also known as the Jaccard Index. These metrics quantify the topological accuracy of the predicted segmentation masks against the manually annotated ground truth. Both metrics rely on the calculation of True Positives (TP), False Positives (FP), and False Negatives (FN) at the pixel level.

The Intersection over Union calculates the ratio of the area of overlap between the predicted and ground truth masks to the area of their union. It is mathematically defined as

(2) $$\mathrm{IoU}=\frac{TP}{TP+FP+FN}$$

This metric heavily penalizes both under-segmentation and over-segmentation. The Dice Similarity Coefficient is defined as the harmonic mean of precision and recall. It is mathematically expressed as

(3) $$\mathrm{DSC}=\frac{2\times TP}{2\times TP+FP+FN}$$

The Dice Similarity Coefficient generally yields a slightly higher numerical value than the Intersection over Union and is particularly effective for evaluating highly imbalanced datasets, such as the thin ring structures of myelin sheaths set against a large background of axoplasm and extracellular matrix.

## 3. Results

### 3.1. Quantitative Evaluation of the Generalized Segmentation Pipeline

To objectively validate the performance of the standardized deep learning pipeline, the models were evaluated in two distinct phases: patch-based algorithmic convergence during training (Table 1) and independent spatial evaluation on continuous tissue regions (Table 2). Performance was quantified using the Intersection over Union (IoU) and the Dice Similarity Coefficient (DSC).

To establish comparative learning capacity, the Generalized models were first evaluated against domain-specific Baseline models operating on raw, un-preprocessed data for highly resolvable targets. As shown in Table 1, the Generalized model achieved robust patch-level spatial overlap across all evaluated ultrastructural classes on the composite dataset during training. While specialist Baseline models achieved high accuracy on their native training patches (e.g., 0.9665 IoU for Caudal myelin), the Generalized model demonstrated consistent convergence across all combined domains simultaneously (0.9535 IoU for myelin; 0.9112 IoU for mitochondria). Furthermore, the Generalized model successfully segmented highly complex micro-organelles, such as the fragmented ER (IoU of 0.8248, DSC of 0.9040), which were challenging to reliably train using Baseline models on raw, unstandardized datasets due to limited local contrast.

To quantify the spatial advantage of the standardization pipeline, the Generalized models were subsequently evaluated against the Baseline models on independent, continuous tissue Regions of Interest (N = 30 per domain). Statistical significance was determined via paired Wilcoxon signed-rank tests (Table 2). This comparative breakdown highlights distinct behavioral differences between macro- and micro-architectural segmentation, supporting the utility of the pre-processing script in domain generalization.

For micro-organelles (mitochondria), the Generalized model demonstrated consistent, statistically significant improvements (*p* < 0.001) across all three nerve domains. Baseline models exhibited reduced performance when transitioning from isolated training patches to continuous tissue regions (e.g., decreasing to 0.552 IoU on the Uranyl-free domain), whereas the Generalized model mitigated this effect, yielding improved spatial accuracy (0.695 IoU, *p* < 0.001).

For macro-structures (myelin), the Generalized pipeline achieved performance parity with the specialized models without requiring domain-specific retraining. It outperformed the Uranyl-free staining Baseline (*p* < 0.001) and performed comparably to the Ellisman Baseline (*p* = 0.543). While the Caudal Baseline achieved a higher pixel-wise IoU on its native domain (0.936 vs. 0.880, *p* < 0.001), visual inspection suggests this metric may be influenced by localized pixel-boundary coupling to the specific training annotation style. The Generalized model, conversely, generated morphologically continuous myelin boundaries. Ultimately, this independent spatial evaluation indicates that the equalization script serves as an important factor for maintaining structural accuracy and generalization across varying imaging domains.

### 3.2. Generalization Capability and Performance

**Figure 1:**
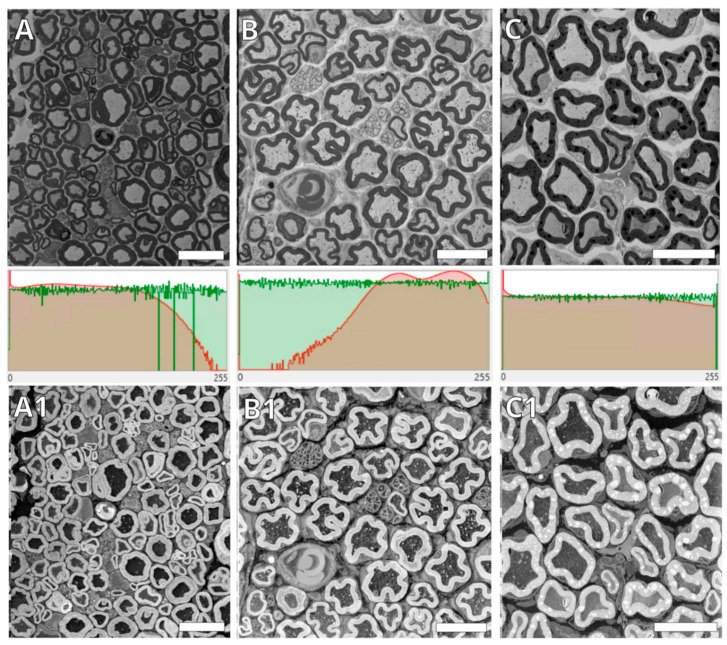
Image standardization and contrast enhancement across diverse electron microscopy nerve tissue preparations. (**A**) Raw, low-resolution sample of a sciatic nerve prepared using the Ellisman method, optimized for myelin sheath examination. (**B**) Raw cross-section of a caudal nerve stained with the Palade method. (**C**) Raw cross-section of a sciatic nerve prepared using a uranyl-free staining method. Histograms (middle row): Pixel intensity distributions (0–255) for the corresponding image columns. Red filled areas denote the highly variable, unequalized distributions of the raw top-row samples (**A**–**C**). Green lines represent the maximized, uniform distributions following equalization. (**A1**–**C1**) The identical tissue samples from the top row (**A**, **B**, and **C**, respectively) after the application of the custom equalization script, exhibiting standardized global contrast and sharply delineated target structures across all domains. Scale bars: 10 µm.

The application of the custom equalization script successfully mitigated the high inter-sample variance caused by diverse preparation protocols, significantly improving image quality for downstream segmentation. While the raw images (Figure 1A–C) exhibited restricted dynamic ranges and highly variable baseline contrasts dependent on their specific stains, the script effectively standardized these photometric profiles.

This improvement is demonstrated by the histogram distributions; the script transformed the skewed, stain-specific intensity peaks of the raw images (represented by the red filled areas) into a uniform, maximized distribution across the full 0–255 pixel range (represented by the green overlaid lines).

Qualitatively, this transformation yielded a substantial enhancement in structural clarity and edge definition across all samples (Figure 1A1–C1). By maximizing the local contrast, the boundaries of the electron-dense myelin sheaths became sharply and consistently delineated from both the internal axoplasm and the surrounding extracellular matrix. Crucially, this improvement in target structure visibility was achieved irrespective of whether the original sample was a low-resolution scan (Figure 1A1) or prepared with different contrasting agents like Palade (Figure 1B1) or Uranyl-free methods (Figure 1C1). Ultimately, the script standardized the distinct visual domains, stripping away confounding lighting and staining variations so that the structural morphology is clearly emphasized.

**Figure 2:**
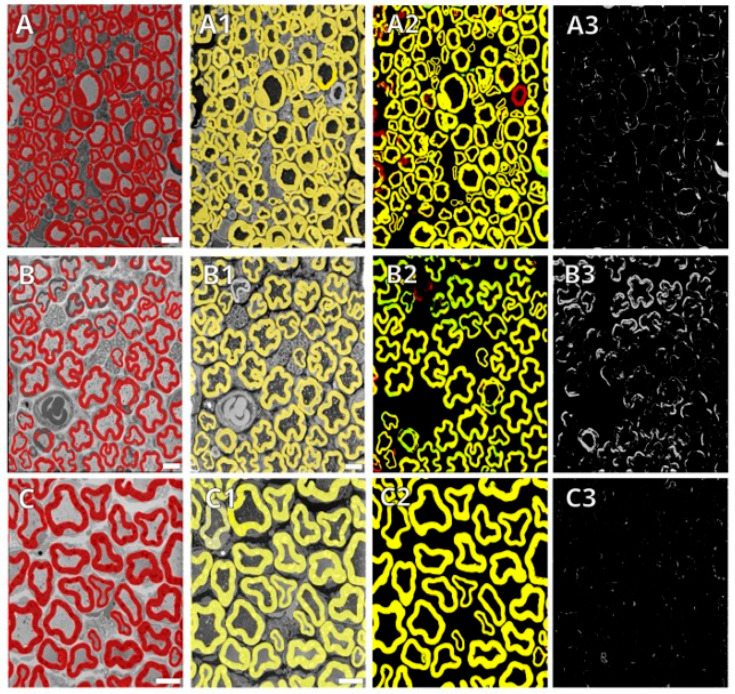
Multi-domain structural evaluation and morphological divergence mapping of myelin segmentation. Representative regions of interest across three distinct nerve preparations are compared: (**Row A**) Ellisman sciatic nerve, (**Row B**) Palade caudal nerve, and (**Row C**) Uranyl-free stained sciatic nerve. (**A**–**C**) Raw, unequalized tissue samples with predictive myelin masks generated by the domain-specific Baseline models overlaid in red. (**A1**–**C1**) Standardized tissue samples with predictive myelin masks generated by the Generalized model overlaid in yellow. (**A2**–**C2**) Direct composite overlays of both the Baseline (red) and Generalized (yellow) masks to visualize spatial consensus. (**A3**–**C3**) Morphological divergence maps (mask subtractions) isolating the precise spatial discrepancies between the two models. In these subtraction maps, regions unique to the Generalized model demonstrate smooth, continuous structural interpolation, whereas regions unique to the Baseline model frequently reflect localized pixel-boundary coupling. Scale bars: 10 µm.

**Figure 3:**
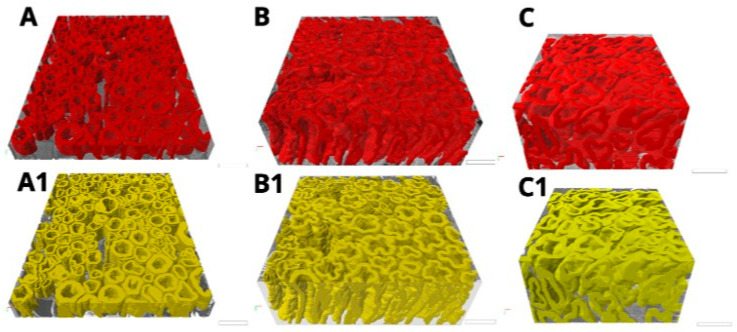
Impact of pre-processing standardization on 3D volumetric reconstruction fidelity. (**A**–**C**) 3D reconstructions derived from sequential 2D segmentations performed on raw, unequalized images, rendered in red. The accumulation of 2D over-segmentation errors results in amorphous, fused volumes that fail to isolate individual fibers. (**A1**–**C1**) 3D reconstructions derived from sequential 2D segmentations performed on standardized images, rendered in yellow. The improved precision of the standardized 2D masks yields continuous, distinct, and biologically accurate tubular myelin structures. Scale bars: 10 µm.

Figure 2 and Figure 3 visually contextualize the performance of the standardized Generalized segmentation pipeline compared to the domain-specific Baseline models, evaluated across both 2D cross-sections and 3D volumetric reconstructions.

In the 2D evaluations (Figure 2), predictive masks generated by the Baseline models (Figure 2A–C; red masks) exhibit strict adherence to the localized pixel variations in their specific training annotations. While this localized pixel-boundary coupling can yield high domain-specific mathematical overlap, it frequently results in jagged, irregular boundary representations. Conversely, segmentations generated by the Generalized model operating on standardized images (Figure 2A1–C1; yellow masks) demonstrate continuous, high-fidelity structural interpolation. The morphological divergence maps (Figure 2A3–C3) isolate these precise spatial discrepancies, highlighting how the Generalized model mitigates localized boundary coupling to produce smooth, continuous myelin sheaths across diverse staining domains.

These morphological distinctions observed in 2D are conserved in the 3D volumetric reconstructions (Figure 3). While both pipelines successfully generate viable macroscopic representations of the tubular myelin sheaths, reconstructions derived from the Baseline segmentations (Figure 3A–C; red volumes) exhibit minor surface irregularities along the longitudinal axis. This reflects the inter-slice accumulation of the localized 2D boundary overfitting. Conversely, 3D reconstructions based on the Generalized model outputs (Figure 3A1–C1; yellow volumes) maintain a higher degree of Z-axis continuity. The reduction in localized 2D artifacts translates into smoother surface topographies, demonstrating that the standardization pipeline effectively reduces domain-specific volumetric noise during large-scale morphological analysis.

### 3.3. Segmentation of Complex Cellular Structures

**Figure 4:**
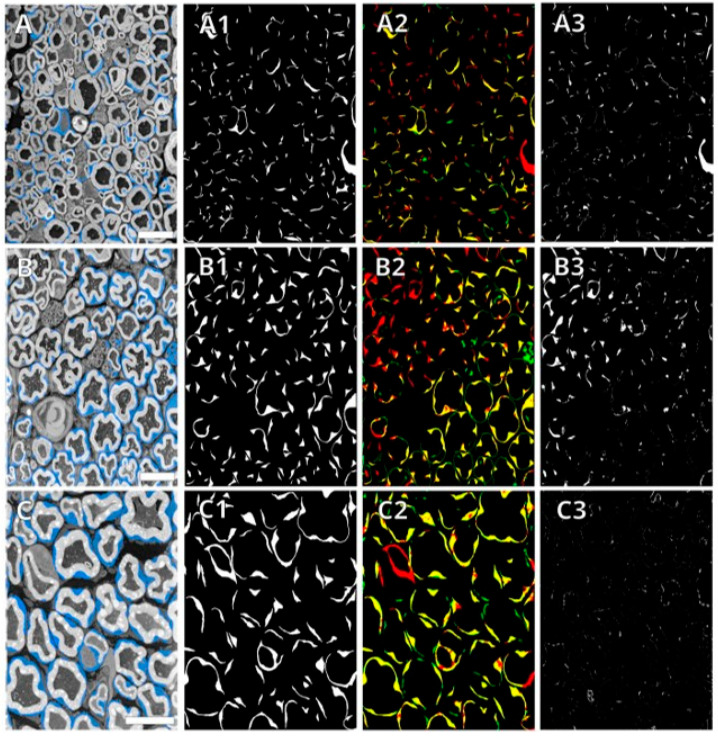
Spatial accuracy evaluation of Generalized Schwann cell segmentation. Representative regions of Schwann cell cytoplasm segmentation evaluated against manually annotated ground truth across three diverse nerve preparations: (**Row A**) Ellisman sciatic nerve, (**Row B**) Palade caudal nerve, and (**Row C**) Uranyl-free stained sciatic nerve. (**A**–**C**) Standardized tissue samples displaying the Generalized model’s spatial predictions overlaid in blue. (**A1**–**C1**) Corresponding manually annotated binary ground truth masks. (**A2**–**C2**) Spatial accuracy maps generated by directly overlaying the ground truth (red) and model prediction (green). In these composites, yellow represents True Positives (spatial consensus), red indicates False Negatives (ground truth structures missed by the model), and green indicates False Positives (model over-segmentation). (**A3**–**C3**) Spatial difference maps generated by subtracting the AI prediction from the ground truth, highlighting the precise boundaries of the segmentation discrepancies. Scale bars: 10 µm.

Schwann cells present an exceptionally difficult segmentation challenge due to their thin, highly irregular cytoplasmic processes that wrap around the myelin sheaths and interdigitate with the surrounding endoneurial collagen. Furthermore, their mid-range grayscale values frequently cause them to blend with the background extracellular matrix or adjacent electron-dense structures in raw preparations. Despite these basal challenges, segmentations generated by the Generalized model operating on the standardized datasets (Figure 4A–C; blue masks) demonstrate robust spatial tracking of this complex geometry across all three staining domains.

Because reliable baseline models could not be trained on un-preprocessed data for these low-contrast targets, the spatial fidelity of the Generalized model was evaluated directly against manually annotated ground truth (Figure 4A1–C1). The resulting spatial accuracy composites (Figure 4A2–C2) reveal a high degree of structural consensus (yellow). Raw images frequently contain confounding ultrastructural elements, such as high-contrast collagen bundles, that mimic cellular boundaries. The spatial accuracy maps demonstrate that the Generalized model generally remains robust to these domain-specific artifacts, reliably distinguishing the fine cellular cytoplasm from the extracellular matrix without severe false-positive over-segmentation.

The isolated spatial difference maps (Figure 4A3–C3) further illustrate the model’s precision, primarily highlighting minor, localized boundary deviations. However, rigorous visual interrogation also reveals specific limitations in areas of extreme morphological ambiguity. For instance, the model struggles with localized boundary adherence in the peripheral region of the Palade-stained caudal nerve (Figure 4B2,B3,top left). This deviation occurs where the cytoplasmic processes become exceptionally attenuated and structurally indistinguishable from the dense surrounding collagen networks, exceeding the current limits of the spatial resolution. Despite these localized regional challenges, the Generalized model maintains high quantitative accuracy in segmenting the associated Schwann cell nuclei (as detailed in Table 1), maintaining strict and morphologically accurate boundaries between the mid-tone cytoplasm, the nucleoplasm, and the surrounding structures. Ultimately, this spatial fidelity confirms that standardizing the input space provides the necessary local contrast to overcome subtle grayscale ambiguities and reliably segment highly complex cellular architectures.

### 3.4. Intracellular Organelle Segmentation

Beyond large macro-structures such as myelin sheaths, we evaluated the Generalized model’s capacity to segment fine subcellular organelles, specifically mitochondria. Mitochondria pose a distinct segmentation challenge due to their small cross-sectional area, variable spatial distribution, and their tendency to blend with the granular texture of the surrounding axoplasm, particularly in low-contrast images.

**Figure 5:**
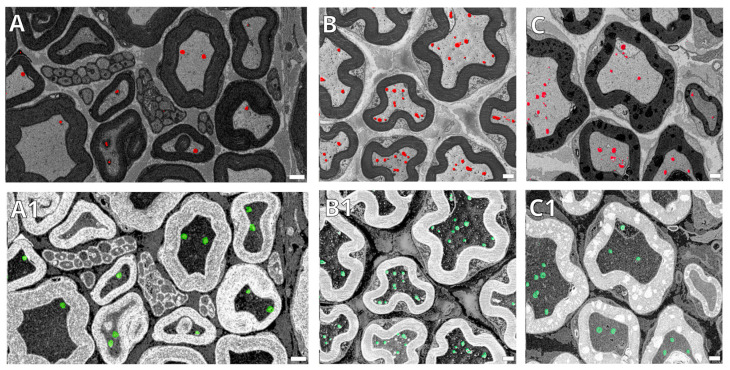
Impact of image standardization on the segmentation of mitochondria across diverse imaging domains. (**A**–**C**) Raw, unequalized electron microscopy images (representing Ellisman, Palade, and Uranyl-free preparations, respectively) with corresponding mitochondrial segmentations overlaid in red. (**A1**–**C1**) The identical tissue samples after the application of the custom equalization script, with mitochondrial segmentations overlaid in light green. The standardized contrast drastically improves the Generalized model’s ability to reliably detect and precisely delineate mitochondria against the axoplasm across all heterogeneous datasets. Scale bars: 10 µm.

Figure 5 illustrates the impact of the pre-processing pipeline on the model’s ability to resolve these structures. When applied directly to the raw, unequalized images (Figure 5A–C; red masks), the model’s performance is hindered by the varying baseline contrasts across the Ellisman, Palade, and Uranyl-free domains. The low intrinsic contrast between the mitochondria and the intra-axonal background makes consistent boundary delineation difficult.

However, the application of the custom equalization script (Figure 5A1–C1; light green masks) significantly enhances the local contrast, effectively isolating the mitochondria from the surrounding axoplasmic noise. By standardizing the photometric profiles across all domains, the Generalized model achieves highly precise and robust segmentations of these small, discrete organelles. The resulting light green masks demonstrate sharp boundary adherence and consistent detection rates across all three disparate tissue preparations, confirming that the harmonization pipeline is critical for the accurate quantification of fine subcellular targets.

The segmentation of the ER within nerve fibers represents a challenging ultrastructural task due to its small physical scale and variable morphology. Unlike larger organelles, axonal ER frequently presents merely as a scattered collection of few-pixel-wide dots or highly fragmented thin tubules distributed sparsely throughout the axoplasm. Furthermore, at mitochondria-associated membrane (MAM) sites, the ER often appears as isolated vesicles, adding significant structural heterogeneity to the target class.

**Figure 6:**
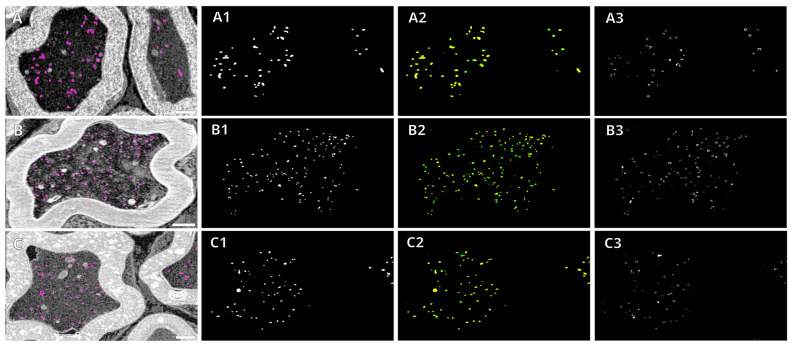
Spatial accuracy evaluation of Generalized endoplasmic reticulum segmentation. Representative regions of fragmented endoplasmic reticulum evaluated against manually annotated ground truth across three diverse nerve preparations: (**Row A**) Ellisman sciatic nerve, (**Row B**) Palade caudal nerve, and (**Row C**) Uranyl-free stained sciatic nerve. (**A**–**C**) Standardized tissue samples displaying the Generalized model’s spatial predictions overlaid in magenta. (**A1**–**C1**) Corresponding manually annotated binary ground truth masks. (**A2**–**C2**) Spatial accuracy maps generated by directly overlaying the ground truth and model prediction. In these composites, yellow represents True Positives (spatial consensus), while red and green indicate localized False Negatives and False Positives, respectively. (**A3**–**C3**) Spatial difference maps isolating the precise boundaries of the segmentation discrepancies. Scale bars: 1 µm.

Because of this extremely small spatial footprint and their mid-range grayscale intensities, ER structures in raw, unstandardized images are highly challenging to distinguish from background axoplasmic noise, staining precipitates, or general image grain. Consequently, attempting to train reliable domain-specific Baseline models directly on raw datasets is unfeasible due to this fundamental lack of basal contrast. To overcome these limitations, the spatial fidelity of the Generalized model for ER segmentation was evaluated exclusively on standardized datasets, measured directly against manually annotated ground truth (Figure 6).

Across the diverse Ellisman (Figure 6A), Palade (Figure 6B), and Uranyl-free (Figure 6C) preparations, the Generalized model predictions (magenta) successfully localize these sparse targets. The spatial accuracy composites (Figure 6A2–C2) reveal a high degree of structural consensus (yellow) with the ground truth (Figure 6A1–C1). Despite the target structures often comprising only a handful of pixels, the isolated spatial difference maps (Figure 6A3–C3) confirm that the model accurately identifies scattered axonal ER and vesicular MAM formations with minimal false-positive over-segmentation in the surrounding standardized axoplasm. This visual evidence reinforces the quantitative training metrics (detailed in Table 1), demonstrating that harmonization is an absolute prerequisite for the deep learning-based mapping of highly fragmented micro-organelles.

### 3.5. Integrated Multi-Class 3D Reconstruction

**Figure 7:**
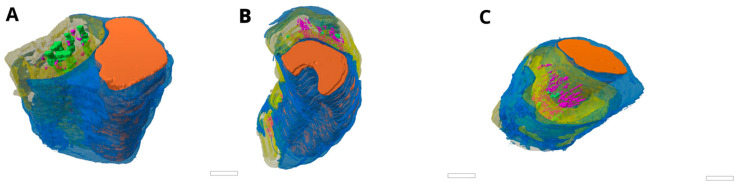
Comprehensive 3D multi-class reconstructions of myelinated nerve fibers across diverse imaging domains. Volumetric models were derived from the standardized datasets to display the spatial relationships between the Schwann cell cytoplasm (blue), Schwann cell nucleus (orange), myelin sheath (yellow), mitochondria (green), and endoplasmic reticulum (magenta). (**A**) Reconstruction from the low-resolution dataset (Ellisman preparation), demonstrating successful organelle recovery from suboptimal tissue. (**B**) Reconstruction from the traditional, high-contrast Palade preparation. (**C**) Reconstruction from the lanthanide-based “green” Uranyl-free preparation. The consistent quality of the integrated volumes across all three preparations demonstrates the generalizability of the automated segmentation pipeline. Scale bars: 10 µm.

To validate the cumulative efficacy of the automated segmentation pipeline, comprehensive, multi-class 3D reconstructions of individual myelinated axons and their associated structures were generated across all three diverse imaging domains (Figure 7). These reconstructions demonstrate the capability of the workflow to seamlessly integrate individually segmented classes, myelin (yellow), Schwann cell cytoplasm (blue), Schwann cell nucleus (orange), mitochondria (green), and ER (magenta), into cohesive volumetric models, regardless of the underlying sample preparation.

The successful extraction of these multi-organelle volumes highlights the robustness of the standardized pre-processing and the Generalized deep learning model. In the classical Palade (Figure 7B) and Uranyl-free (Figure 7C) datasets, the pipeline reliably captures both macro-structures and fine subcellular details despite their fundamentally different baseline textures and contrast profiles. Most notably, the pipeline successfully generated a comparable reconstruction from the Ellisman preparation (Figure 7A). Because this protocol is traditionally optimized only for myelin examination and is inherently suboptimal for preserving fine micro-organelles, the ability to reconstruct complex targets like the ER from this sample underscores the critical role of the equalization script in recovering usable biological signals from challenging data.

While the standardized 2D segmentations provided a highly accurate foundation across all domains, rendering fully cohesive 3D volumes of this complexity still presents minor spatial challenges. In particular, ensuring the inter-slice continuity of minute, highly fragmented structures like the ER occasionally required localized spatial curation to resolve alignment artifacts. Nevertheless, these comprehensive 3D models demonstrate that the proposed pipeline effectively bridges the gap between disparate 2D imaging domains and high-fidelity 3D ultrastructural analysis.

### 3.6. Zero-Shot Generalization on Independent Hold-Out Data

To rigorously evaluate generalization capacity, the standardized model was deployed on an independent, unseen 1000-image volumetric stack. This specific dataset consisted of peripheral sciatic nerve tissue derived from a healthy Balb/c mouse, prepared via the standard Ellisman protocol. Crucially, while it shares the macro-biological and chemical domain of the primary training set, it represents a completely separate acquisition batch, providing a true zero-shot test.

**Figure 8:**
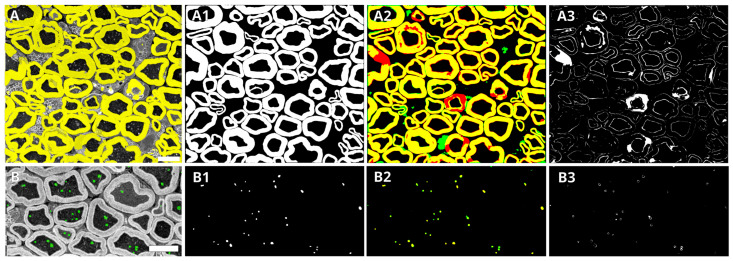
Zero-shot spatial accuracy evaluation on a structurally novel dataset. The generalization capacity of the standardized segmentation pipeline was evaluated on an entirely unseen block-face volume of an Ellisman-prepared sciatic nerve. Representative cross-sections demonstrate zero-shot spatial tracking for (**Row A**) myelin sheaths and (**Row B**) mitochondria. (**A**,**B**) Standardized unseen tissue samples displaying the Generalized model’s spatial predictions overlaid in yellow (myelin) and green (mitochondria). (**A1**,**B1**) Corresponding manually annotated binary ground truth masks. (**A2**,**B2**) Spatial accuracy maps generated by directly overlaying the ground truth and model prediction. In these composites, yellow represents True Positives (spatial consensus), while red indicates False Negatives and green indicates False Positives. (**A3**,**B3**) Spatial difference maps isolating the precise boundaries of the segmentation discrepancies. Scale bars: 5 µm.

To appropriately evaluate structures across different biological scales, testing was performed on distinct regions of interest acquired at a baseline voxel resolution of 10 nm × 10 nm × 50 nm. Macroscopic myelin sheath segmentation was evaluated on a wide-field 8000 × 3771 pixel region, while intracellular mitochondria were evaluated within targeted 2993 × 1564 pixel sub-crops to preserve high-magnification structural fidelity. To ensure a comprehensive evaluation across the entire Z-axis, forty representative slices (N = 40) were selected using a stratified sampling approach: 5 slices from the top, 30 from the middle, and 5 from the bottom of the 1000-image stack. This intentional inclusion of the volume’s upper and lower extremities rigorously tests the model against structural “edge cases”—regions where block-face surface artifacts, anisotropic stretching, and contrast degradation are typically most severe. Prior to inference, these images were passed through the identical preprocessing pipeline (NLM-denoising, Z-score normalization, and CLAHE) described in Section 2.2, ensuring the model operated exclusively within its trained, standardized feature space (Figure 8).

For macroscopic myelin sheath segmentation (Figure 8, Row A), the model demonstrated exceptional morphological fidelity across the expanded validation set, achieving an average Dice Similarity Coefficient (DSC) of 0.981 ± 0.017 and an Intersection over Union (IoU) of 0.963 ± 0.032. Visual inspection of the model predictions (Figure 8A), manual ground truth (Figure 8A1), and the resulting spatial accuracy composite (Figure 8A2) confirms this robust performance, characterized by near-total spatial consensus (yellow). The isolated difference map (Figure 8A3) further verifies that spatial discrepancies are restricted to minor, sub-pixel boundary deviations along the inner and outer myelin perimeters.

Crucially, this zero-shot generalization extended to the detection of small intracellular organelles. For axoplasmic mitochondria (Figure 8, Row B), the model achieved a highly robust DSC of 0.857 ± 0.070 (IoU: 0.756 ± 0.096). While these numerical intersection metrics are naturally slightly lower than those of macroscopic structures, the spatial accuracy and difference maps (Figure 8B2,B3) confirm precise biological target localization. This variance is an inherent mathematical artifact of the exceedingly small pixel-footprint of these organelles; minor boundary-width discrepancies disproportionately penalize the IoU, even when the model successfully detects the organelle without systemic false negatives or widespread false positives. Collectively, these hold-out metrics confirm the pipeline’s robust zero-shot generalization, demonstrating that standardizing the input space enables stable, automated ultrastructural mapping across highly variable Z-axis without the need for continuous retraining.

## 4. Discussion

The performance of the Generalized model offers a robust counterpoint to the common assumption in bioimage analysis that domain-specific specialist models inherently perform best. As demonstrated in our 2D and 3D evaluations, Baseline models trained exclusively on raw data severely overfit to localized pixel noise, resulting in jagged, domain-coupled boundary predictions. Conversely, our results suggest that combining diverse datasets, when coupled with rigorous spatial standardization, serves as a highly effective form of domain randomization [21]. By training the neural network across three distinct visual styles to recognize the fundamental morphological concepts of diverse targets, ranging from macroscopic myelin sheaths to fine micro-organelles, the Generalized model becomes decoupled from specific textural cues, such as the exact shade of gray produced by uranium binding. Instead, the model learns robust, invariant geometric features [22], enabling continuous, biologically plausible structural interpolation even in areas of ambiguous basal contrast.

While deep learning provides the segmentation engine, the custom equalization script integrated into the workflow plays a critical role in facilitating this biological interpolation. Convolutional neural networks are inherently sensitive to input distribution shifts [13,14]. By normalizing the histogram, balancing the dynamic range, and applying targeted enhancements prior to the inference stage, the script successfully minimizes the domain gap between a traditional heavy-metal stain and an alternative preparation. This approach supports a hybrid workflow that utilizes classical computational image processing to standardize data for artificial intelligence applications [23]. Furthermore, while state-of-the-art architectures like nnU-Net represent the performance benchmark for medical image segmentation, our pipeline prioritizes seamless integration within a unified, commercially supported environment (ZEISS Arivis Pro). This eliminates the need for complex command-line deployment and cross-platform data migration, streamlining the transition from image acquisition to volumetric analysis.

These findings hold promising implications for the future of neuropathological analysis. The pipeline helps lower the computational barrier to adopting “green” staining methods, assisting laboratories in transitioning to safer, non-toxic chemicals like uranyl-free protocols. Crucially, our results challenge the historical assumption that non-toxic alternatives represent an imaging compromise. In fact, the uranyl-free preparations exhibited outstanding basal contrast for cellular membranes and organelle boundaries. When standardized through our pre-processing pipeline, the generalized model leveraged this strong inherent signal to achieve near-perfect segmentation (IoU: 0.992). This conclusively demonstrates that laboratories can adopt safer, heavy-metal-free protocols while actually maximizing the accuracy of downstream automated analysis. Importantly, this entire pipeline was executed on a standard workstation equipped with a single NVIDIA RTX 4000 GPU (8 GB VRAM). Unlike compute-heavy models requiring server-grade clusters, this hardware efficiency democratizes advanced organellomics, making it highly accessible for standard biology laboratories. Additionally, the ability to segment complex morphologies, such as interdigitating Schwann cells and minute endoplasmic reticulum vesicles, suggests that historical archives of unstandardized legacy data could be more readily utilized for quantitative, high-throughput analysis.

A critical strength of this standardized approach is its stability during zero-shot validation on novel datasets. Unlike typical deep learning deployments where open-world performance drops precipitously compared to internal training metrics, our expanded zero-shot hold-out (N = 40) demonstrated exceptional stability (Myelin IoU: 0.963). For smaller intracellular structures like mitochondria, the zero-shot IoU of 0.756 represents highly robust spatial target localization. As visualized in the spatial difference maps (Figure 8), variances in mitochondrial segmentation are strictly confined to sub-micron boundary width mismatches rather than missed organelles or false-positive hallucinations. This aligns perfectly with recent methodological consensus demonstrating that overlap-based metrics like IoU and Dice disproportionately penalize minor boundary variances in small-scale objects, artificially suppressing mathematical scores even when spatial localization remains biologically accurate [24].

Despite this promising performance, several limitations of the current study must be acknowledged. First, the models were trained exclusively on tissue derived from healthy Balb/c mice. Consequently, the pipeline’s performance on pathological samples remains untested. In advanced neuropathies, organelles frequently exhibit significant structural alterations, such as pronounced mitochondrial swelling or complex myelin outfoldings, that deviate from the geometric baselines established in our healthy dataset. Future development will therefore require testing on diverse pathological models. Additionally, while the model demonstrated high spatial fidelity for Schwann cell nuclei (Table 1), the natural scarcity of these structures within 2D cross-sections inherently limits the statistical power of this specific evaluation. Expanded volumetric datasets will be required to capture a statistically robust sample size of these sparse nuclear events. Furthermore, acquiring fully independent, annotated 3D volumes for the Uranyl-free and Palade protocols remains a necessary future objective to comprehensively evaluate the pipeline’s generalization capabilities.

From a technical perspective, the deep learning inference currently operates on 2D cross-sections. While highly effective for planar morphological features, it presents limitations in areas of extreme structural ambiguity. For instance, as observed in the Palade caudal nerve (Figure 4B2), the model occasionally struggles with localized boundary adherence when highly attenuated Schwann cell processes become tangentially sectioned and structurally indistinguishable from dense surrounding collagen networks in 2D space. Assembling these sequential 2D masks into cohesive 3D volumes inherently carries over these planar limitations. Additionally, the CLAHE-based pre-processing script has inherent bounds; in regions of extreme low signal or empty resin, aggressive histogram equalization can inadvertently over-amplify background noise, requiring careful thresholding. Furthermore, while our deployment within ZEISS Arivis Pro provides a highly streamlined, code-free environment for biologists, we acknowledge that this reliance on a commercial platform represents a notable limitation for open-source reproducibility, though the fundamental pre-processing and domain-randomization principles remain framework-agnostic. Finally, the pipeline’s zero-shot generalization capabilities to fundamentally unseen imaging modalities outside the peripheral nervous system remain untested.

Future development will focus on addressing these spatial constraints. A logical next step is the transition from 2D slice-by-slice segmentation to native 3D volumetric deep learning architectures. Leveraging spatial context across the z-axis could help resolve extreme peripheral entanglements and inter-slice ambiguities automatically, potentially reducing the reliance on manual alignment for complex 3D reconstructions. Additionally, subsequent studies should explore expanding the multi-domain training dataset to encompass a wider variety of tissue types and alternative imaging modalities, such as focused ion beam scanning electron microscopy (FIB-SEM). This expansion would help determine the broader applicability of this domain-randomization approach across the wider field of computational pathology.

## 5. Conclusions

This study presents a robust computational pipeline for the automated segmentation of peripheral nerve and ganglia ultrastructure. By integrating a custom Python pre-processing workflow into the Arivis Pro environment and employing a multi-domain training strategy, we developed deep learning models that demonstrate high zero-shot adaptability across different staining chemistries and image resolutions, validated across a comprehensive testing cohort (N = 40). To ensure transparency and broad community utility, the pre-trained Generalized models and pre-processing scripts have been made open-access via a public repository, removing the barrier of commercial cloud-subscription requirements. While this specific implementation utilizes the proprietary Arivis Pro ecosystem, the underlying principles of computational standardization and domain randomization are framework-agnostic. Ultimately, this approach facilitates the seamless transition to alternative electron microscopy protocols and significantly reduces the frequent need to retrain models for individual datasets.

## Figures and Tables

**Table 1:** Network Architecture and Patch-Based Training Metrics. Note: IoU values represent internal validation computed dynamically by the Arivis software on isolated image patches during training (Best Epoch), not whole-image continuous spatial evaluation.

Target Structure	Dataset Domain	Model Type	IoU (Jaccard)	Dice Score (DSC)
Myelin Sheath	Caudal Nerve (Palade)	Baseline (Raw)	0.9665	0.9830
	Sciatic Nerve (Uranyl-free)	Baseline (Raw)	0.9513	0.9750
	Clinical (Ellisman)	Baseline (Raw)	0.9430	0.9707
	All Domains Combined	Generalized (Standardized)	0.9535	0.9762
Mitochondria	Caudal Nerve (Palade)	Baseline (Raw)	0.8899	0.9417
	Sciatic Nerve (Uranyl-free)	Baseline (Raw)	0.8106	0.8954
	Clinical (Ellisman)	Baseline (Raw)	0.8028	0.8906
	All Domains Combined	Generalized (Standardized)	0.9112	0.9535

**Table 2:** Independent Spatial Evaluation (Internal Domain Cohorts). Note: Independent evaluation performed on continuous Regions of Interest (ROIs). Significance calculated via paired Wilcoxon signed-rank test (N = 30 slices per domain).

Nerve Domain	Structure	Baseline Model (Mean IoU ± SD)	Universal Model (Mean IoU ± SD)	Wilcoxon *p*-Value
Uranyl-free (sciatic)	Myelin	0.968 ± 0.002	0.992 ± 0.001	*p* < 0.001 (Generalized)
	Mitochondria	0.552 ± 0.040	0.695 ± 0.040	*p* < 0.001 (Generalized
Pallade (caudal)	Myelin	0.936 ± 0.016	0.880 ± 0.029	*p* < 0.001 (Baseline)
	Mitochondria	0.618 ± 0.021	0.692 ± 0.042	*p* < 0.001 (Generalized)
Ellisman (sciatic)	Myelin	0.918 ± 0.008	0.917 ± 0.013	*p* = 0.543 (NS)
	Mitochondria	0.634 ± 0.021	0.723 ± 0.024	*p* < 0.001 (Generalized)

(NS = Not Significant. Note: As discussed in text, higher strict IoU for Caudal Baseline Myelin reflects localized boundary overfitting, whereas the Universal model achieved superior morphological continuity).

## Data Availability

The custom Python-based pre-processing script developed for this study and the models are publicly available on GitHub at https://github.com/Nochebald/EM-raw-image-standardization.git (accessed on 2 June 2026). The raw electron microscopy datasets are available from the corresponding author upon reasonable request.

## References

[1] Tsutsumi Y. (2018). Electron Microscopic Study Using Formalin-Fixed, Paraffin-Embedded Material, with Special Reference to Observation of Microbial Organisms and Endocrine Granules. Acta Histochem. Cytochem..

[2] Pareyson D., Marchesi C. (2009). Diagnosis, Natural History, and Management of Charcot-Marie-Tooth Disease. Lancet Neurol..

[3] Smith S., Normahani P., Lane T., Hohenschurz-Schmidt D., Oliver N., Davies A.H. (2022). Pathogenesis of Distal Symmetrical Polyneuropathy in Diabetes. Life.

[4] Kaiser T., Allen H.M., Kwon O., Barak B., Wang J., He Z., Jiang M., Feng G. (2021). MyelTracer: A Semi-Automated Software for Myelin g-Ratio Quantification. eNeuro.

[5] Viader A., Golden J.P., Baloh R.H., Schmidt R.E., Hunter D.A., Milbrandt J. (2011). Schwann Cell Mitochondrial Metabolism Supports Long-Term Axonal Survival and Peripheral Nerve Function. J. Neurosci..

[6] Zhang Q., Song W., Zhao B., Xie J., Sun Q., Shi X., Yan B., Tian G., Liang X. (2021). Quercetin Attenuates Diabetic Peripheral Neuropathy by Correcting Mitochondrial Abnormality via Activation of AMPK/PGC-1α Pathway in Vivo and in Vitro. Front. Neurosci..

[7] Conrad R., Narayan K. (2023). Instance Segmentation of Mitochondria in Electron Microscopy Images with a Generalist Deep Learning Model Trained on a Diverse Dataset. Cell Syst..

[8] Bermudez-Chacon R., Altingovde O., Becker C., Salzmann M., Fua P. (2020). Visual Correspondences for Unsupervised Domain Adaptation on Electron Microscopy Images. IEEE Trans. Med. Imaging.

[9] Palade G.E. (1952). A Study of Fixation for Electron Microscopy. J. Exp. Med..

[10] Moscardini A., Di Pietro S., Signore G., Parlanti P., Santi M., Gemmi M., Cappello V. (2020). Uranium-Free X Solution: A New Generation Contrast Agent for Biological Samples Ultrastructure. Sci. Rep..

[11] Kuipers J., Giepmans B.N.G. (2020). Neodymium as an Alternative Contrast for Uranium in Electron Microscopy. Histochem. Cell Biol..

[12] Friedman P.L., Ellisman M.H. (1981). Enhanced Visualization of Peripheral Nerve and Sensory Receptors in the Scanning Electron Microscope Using Cryofracture and Osmium-Thiocarbohydrazide-Osmium Impregnation. J. Neurocytol..

[13] Litjens G., Kooi T., Bejnordi B.E., Setio A.A.A., Ciompi F., Ghafoorian M., van der Laak J.A.W.M., van Ginneken B., Sánchez C.I. (2017). A Survey on Deep Learning in Medical Image Analysis. Med. Image Anal..

[14] Hesamian M.H., Jia W., He X., Kennedy P. (2019). Deep Learning Techniques for Medical Image Segmentation: Achievements and Challenges. J. Digit. Imaging.

[15] Ma J., He Y., Li F., Han L., You C., Wang B. (2024). Segment Anything in Medical Images. Nat. Commun..

[16] Archit A., Freckmann L., Nair S., Khalid N., Hilt P., Rajashekar V., Freitag M., Teuber C., Spitzner M., Tapia Contreras C. (2025). Segment Anything for Microscopy. Nat. Methods.

[17] Isensee F., Jaeger P.F., Kohl S.A.A., Petersen J., Maier-Hein K.H. (2021). nnU-Net: A Self-Configuring Method for Deep Learning-Based Biomedical Image Segmentation. Nat. Methods.

[18] Borisovs V., Bossi M., Matino L., Marmiroli P., Cavaletti G. (2025). New Approaches Based on Serial-Block Face Electron Microscopy to Investigate the Peripheral Nervous System. J. Peripher. Nerv. Syst..

[19] Borisovs V., Bossi M., Cavaletti G. (2026). Morphometric Analysis of Axonal Ultrastructure: Coordinated Scaling of Organelles and Myelin. Micron.

[20] Zuiderveld K. (1994). Contrast Limited Adaptive Histogram Equalization. Graphics Gems.

[21] Tobin J., Fong R., Ray A., Schneider J., Zaremba W., Abbeel P. Domain Randomization for Transferring Deep Neural Networks from Simulation to the Real World. Proceedings of the 2017 IEEE/RSJ International Conference on Intelligent Robots and Systems (IROS).

[22] Stringer C., Wang T., Michaelos M., Pachitariu M. (2021). Cellpose: A Generalist Algorithm for Cellular Segmentation. Nat. Methods.

[23] Nešić N., Heiligenstein X., Zopf L., Blüml V., Keuenhof K.S., Wagner M., Höög J.L., Qi H., Li Z., Tsaramirsis G. (2024). Automated Segmentation of Cell Organelles in Volume Electron Microscopy Using Deep Learning. Microsc. Res. Tech..

[24] Reinke A., Tizabi M.D., Baumgartner M., Eisenmann M., Heckmann-Nötzel D., Kavur A.E., Rädsch T., Sudre C.H., Acion L., Antonelli M. (2024). Understanding Metric-Related Pitfalls in Image Analysis Validation. Nat. Methods.

